# Human leukocyte antigen class I, class II, and tumor necrosis factor-alpha polymorphisms in a healthy elder Mexican Mestizo population

**DOI:** 10.1186/1742-4933-2-13

**Published:** 2005-11-03

**Authors:** Elena Soto-Vega, Yvonne Richaud-Patin, Luis Llorente

**Affiliations:** 1 Department of Immunology and Rheumatology, Instituto Nacional de Ciencias Médicas y Nutrición Salvador Zubirán. Mexico City, Mexico

## Abstract

**Background:**

There is strong evidence that an individual's genetic background is an important predisposing factor to longevity. In the present study we analysed the frequency of HLA class I, class II, as well as the TNF-α -308 polymorphism that may be related to an increased life span in Mexican Mestizo healthy elders.

**Results:**

HLA typing was performed by polymerase chain reaction sequence specific oligonucleotide (PCR SSO) reverse dot blot. The TNF-α -308 polymorphism was assessed by PCR restriction fragment length polymorphism. A significant increased frequency of HLA-DRB1*11 was found in elderly women whereas this allele was not present in elderly males. The TNF2 allele was also increased in the elder group when compared to young controls. The frequencies of the remaining alleles tested were not statistically different among groups.

**Conclusion:**

These data suggest an ethnicity independent tendency of HLA-DRB1*11 in elder females to increase life span and a possible role of the TNF2 allele with the successful remodelling of senescent immune system.

## Background

Ageing is associated with a complex remodelling of the immune system often in the direction of apparently decreased immune competence. However, it has been proposed that longevity is related to optimal function of the immune system because some genetic determinants for successful ageing might reside in those polymorphisms of genes that regulate immune responses e.g. major histocompatibility complex (MHC) [1,2]. The studies of life span in humans have resulted in different, and even controversial, associations of HLA class I, class II and class III genes with old age. Thus, longevity has been shown to be related with the selection of HLA alleles or haplotypes being the more frequently associated the A1 B8 Cw7 DR3 in Caucasians [3,4], the increased or decreased frequency of HLA-B40 [5-7], the augmented frequency of HLA-DR11 in elder women in Caucasian as well as in Japanese populations [8,9] among other alleles. Immunogenetic studies in aged, healthy Mexican Mestizo population have not been yet performed.

Mexican Mestizo individuals have a proportion of 56% Native American Indian genes, 40% Caucasian genes, and 4% African genes [10]. Mestizo population represents a complex mixture of European and American native inhabitants, and constitute the core of the Mexican population. This complex genetic process began in the 16^th ^century and has been expanded during the course of time and continues to be a dynamic process, e.g., African population inhabited the Americas almost 200 years later. On the other hand, aged people in the Mexican population have increased from a life expectancy of 63 years in the 1970's up to 70 years nowadays.

Healthy elderly people show a pro-inflammatory phenotype with increased levels of cytokines such as TNF-α [11]. The TNF-α gene is located within the HLA class III region. This cytokine is involved in tissue remodelling, epithelial cell barrier permeability, macrophage activation, recruitment of inflammatory infiltrate, and up-regulation of adhesion molecules, among other functions. TNF-α also determines the strength, effectiveness and duration of local and systemic inflammatory responses [12].

TNF-α promoter contains numerous polymorphic sites, which are possible targets for transcription factors. The polymorphism at position -308 is defined by de substitution of a G by an A, where the presence of G defines the common allele TNF1 and A defines the TNF2 variant, which is less frequent [13]. Some studies have shown that individuals bearing the TNF2 polymorphism are higher TNF-α producers than those who bear the TNF1 variant [14].

The aim of this study was to evaluate the class I, class II HLA genotypes as well as the TNF-α -308 polymorphism with successful ageing in Mexican Mestizo.

## Results and Discussion

Of the 71 elders, 38 (53.5%) were females and 33 (46.5%) males. Results of the observed and expected antigen and genotype frequencies for HLA-B, -DRB1 and -DQB1 loci were consistent with those predicted by the Hardy-Weinberg equilibrium. It is worth mentioning that the frequencies identified in our population are similar from those reported in previous studies performed in Mexican Mestizo populations [10].

Gene frequencies at the HLA-DRB1 locus were not statistically significant different among groups. The most frequent allele in both, young subjects and elders, was the HLA-DRB1*04. The high resolution typing revealed that the more frequent alleles in the elderly group were HLA-DRB1*0802, DRB1*0701, DRB1*0404 and DRB1*0407, whereas HLA-DRB1*0802, DRB1*0701, DRB1*1101 and DRB1*0404 were the more frequent in the young group, being these alleles not statistically significant.

When the differences between aged males and females were analysed, a decreased frequency of HLA-DRB1*11 in males was found when compared to females, for no one in the former group was HLA-DRB1*11 (p = 0.002; OR = 0.18; CI 95% = 0.06–0.53; p = 0.024 by Bonferroni's correction). On the other hand, when elderly males were compared to young males for HLA-DRB1*11, a diminished frequency was found although not statistically significant when Bonferroni's correction was applied. The high resolution typing showed a decreased frequency of the HLA-DRB1*1101 allele in elderly females when compared to the other HLA-DRB1*11 alleles from young women and, again, this difference was not significant by Bonferroni's correction.

Our data must be balanced against the small number of subjects studied. However, we found that, interestingly, no old man was HLA-DRB1*11, which may suggest that survival being gender-dependent [15,16]. This finding for Mexican Mestizo women is in agreement with previous reports in different populations, which might indicate that the contribution of this allele to an increase in the life span is independent of ethnicity [8].

Concerning the HLA-B and HLA-DQB1 alleles, no differences were observed among elders and young controls nor when compared by gender. The high resolution typing of HLA-DQB1 showed that in the elderly group the most frequent subtypes were HLA-DQB1*0302, DQB1*0402 and DQB1*0501 whereas in the young group were the HLA-DQB1*0302, DQB1*0301, DQB1*0402 and DQB1*0201. Finally, the generic typing of the HLA-B locus showed that the more frequent alleles in both groups were HLA-B*35 and HLA -B*15. A decreased tendency in the HLA-B*14 frequency in the elder group was also observed when compared to that of the control group, although this was not statistically significant.

The analysis of the TNF-α -308 promoter polymorphism showed a diminished frequency of the TNF1 allele accompanied by an increased one in the TNF2 allele in elders. The heterozygote genotype TNF1/2 was more common in the elderly group (9.02%) than in the control group (1.5%) (Table 1). Serum levels of TNF-α showed a slight increase in the elderly group although this difference was not statistically significant (data not shown). Interestingly, it was found that 58% of the TNF2 individuals were HLA-DR3 and HLA-DR4. The -308A TNF-α polymorphism has been shown to vary between individuals. These variants have been associated with certain HLA-DR alleles, e.g., HLA-DR2 subjects are considered low TNF- producers while those bearing HLA-DR3 and HLA-DR4 alleles produce high levels of this cytokine [13,14,17-19]

**Table 1:** TNF-α -308 polymorphism in elderly and young adults.

	**Healthy elders (n = 72)**	**Young controls (n = 198)**	**Statistics**		
	**gf (n = 144)**	**gf (n = 110)**	**P**	**OR**	**CI 95%**
**TNF1**	0.909	0.985	NS	-	-
**TNF2**	0.090	0.015	0.001	0.33	0.14–0.73

gf = gene frequency; OR = Odds ratio; CI = Confidence interval; NS: non significant.

The TNF2 genotype has been related to an increased cytokine transcription rate [20]. Furthermore, it has been proposed that individuals who are heterozygous for this polymorphism possess an optimal inflammatory response that protects them against age-related neurodegeneration [21]. Although the inflammatory process has been related to chronic illnesses, incapacity, and death, it is interesting that in healthy old people this pro-inflammatory phenotype may be involved in the remodeling of the cytokine network which contributes to the successful ageing process [22]. Notwithstanding, lack of such association has also been reported [23,24].

## Conclusion

Longevity studies must ideally include healthy centenarians, as they represent the extreme of human old age. Most studies carried out on centenarians come from either Europe or Asiatic countries such as Japan. It is precisely in these regions that the largest numbers of centenarians can be found. This is closely related on the one hand, with the social, cultural, and economic conditions of developed countries and, on the other, with biological factors including the long history of their ethnic groups, which has permitted a natural selection of genes favouring longevity [25]. By contrast, the ethnic group known as Mexican Mestizo is, in evolutionary terms, a recent one barely 500 years old. This lapse is certainly not enough to establish an allele selection or, even, modify completely the structure of previous haplotypes by the genetic admixture process that could account to change certain HLA clusters of Mexican natives. On the other hand, infectious diseases imported by conquerors indeed, have influenced the selection of HLA repertoire in our region. The cultural and socio-economic conditions of our geographical zone (Mexico and Central America) have contributed to a marked increase in life expectancy, particularly over the last 50 years, even though this is still far removed from that of developed countries. It is worth mentioning that the mean age of our study group is at least 10 years greater than the current life expectancy for Mexico and, hence, they represent the oldest population that have enjoyed successful ageing within our country.

Certainly, a great diversity of genes and factors influence successful ageing. Single nucleotide polymorphisms located in cytokine gene promoters have been demonstrated to affect the binding of transcription factors and, hence, its gene expression. Genetic variants that determine an increased production of anti-inflammatory cytokines or a decreased one of pro-inflammatory cytokines have been associated with successful ageing, suggesting a role in the control of the inflammatory state in the attainment of increased life span [12,26,27].

Moreover, the study of polymorphic genes on the X chromosome do not have to be procrastinated for there are some – albeit relatively old – evidence that they are critical in the genetic regulation of the immune response and thus could be of utmost importance for conditioning the life span expectancy [28-30].

## Methods

### Subjects

A total of 71 healthy elders were studied, age ranged from 80 to 96 years (mean 86.2 years). The control samples were obtained from 99 young (from 21 – 54 years; mean 35.2 years) healthy individuals unrelated to elders. All subjects were Mexican Mestizo which is defined as an individual who was born in Mexico and is descendent from mixed racial ancestry of the native Americans of such region with individuals from Europe (mainly Spain) or Africa, all of them living in Mexico City. A complete social and medical history and physical examination was performed. Laboratory tests included complete blood cell count, erythrocyte sedimentation rate (Westergreen), immunoglobulin levels (IgG, IgA, IgM), serum electrolytes, glucose, creatinine, urea, alkaline phosphatase, aspartate transaminase, total bilirubin, and total cholesterol. All subjects were informed about the objectives and methods of the study. Work has been done according to current law.

### DNA isolation

Genomic DNA was extracted from 5 mL of peripheral whole blood employing the Wizard genomic DNA purification kit (Promega, Madison, WI) according to manufacturer's instructions. Isolated DNA was quantified by spectrophotometry and adjusted to a concentration of 100 ng/μL, and stored at -70°C until use.

### HLA Typing

Generic HLA-DRB1, DQB1, and HLA-B typing was performed by polymerase chain reaction sequence specific oligonucleotide (PCR-SSO) reverse dot blot using the Dynal RELI SSO system (Hoffman-La Roche Ltd. and Roche Molecular Systems, Inc., Alameda, CA) as described previously [31]. High resolution typing of HLA-DRB1 and DQB1 loci was done by sequence based typing method (SBT). The primers' sequences and PCR conditions for amplification of polymorphic exons were obtained from protocols of the 13^th ^International Histocompatibility Workshop (Seattle, 2002).

### TNF-α^-308 ^polymorphism

Genotyping for the TNF-α -308 polymorphism was performed using a PCR fragment amplified using the forward primer 5'-AGG CAA TAG GTT TTG AGG GCC AT-3' and the reverse primer 5'-TCC TCC CTG CTC CGA TTC CG-3' to create a restriction site for the *NcoI *endonuclease (Fermentas, Hanover, MD) according to previous reports [32]. Digestion products were analysed by photo typing in 2% agarose gels and stained with ethidium bromide. Only 55 healthy young subjects were analysed for this polymorphism.

### TNF-α serum levels

Serum from each subject was obtained from 5 mL whole blood and stored at -70°C until tested. An ELISA kit was used for the measurement of TNF-α levels according to manufacturer's instructions (R&D Systems, Minneapolis, MN).

### Statistics

Allele frequencies were evaluated by gene count and 2 × 2 contingency tables. Statistical differences of allele frequencies between elders and controls were done employing chi square test and Yate's correction. Obtained *P *values were subjected to Bonferroni's correction. Odds ratio was calculated for healthy elders' carriers of specific alleles. Data were tested for the goodness of fit between the observed and expected genotype values and their fit to Hardy-Weinberg equilibrium.

## List of Abbreviations

HLA Human Leukocyte Antigen

TNF Tumor Necrosis Factor

PCR Polymerase Chain Reaction

PCR SSO Polymerase Chain Reaction Sequence Specific Oligonucleotide
